# Salvaging a Disaster: “Threading the Wrong Needle”

**DOI:** 10.1016/j.jscai.2023.101115

**Published:** 2023-08-05

**Authors:** Brian Cothern, Michael Kourany, Gregory Elsner, Sina Moainie, James Hermiller

**Affiliations:** a Division of Interventional Cardiology, Ascension St. Vincent, Indianapolis, Indiana; b Division of Cardiothoracic Surgery, Ascension St. Vincent, Indianapolis, Indiana

**Keywords:** complication, Impella, Intuity valve, transcatheter aortic valve replacement

Impella-assisted percutaneous coronary intervention (PCI) in selected high-risk patients received a 2b recommendation in the 2021 American College of Cardiology/American Heart Association/Society for Cardiovascular Angiography & Interventions Coronary Revascularization Guideline.^1^ Due to the large sheath sizes required, bleeding and vascular complications are the most frequent adverse outcomes associated with mechanical support.^2^ However, we present the case of an unusual Impella deployment complication that was successfully treated.

## Case

**Figure 1:**
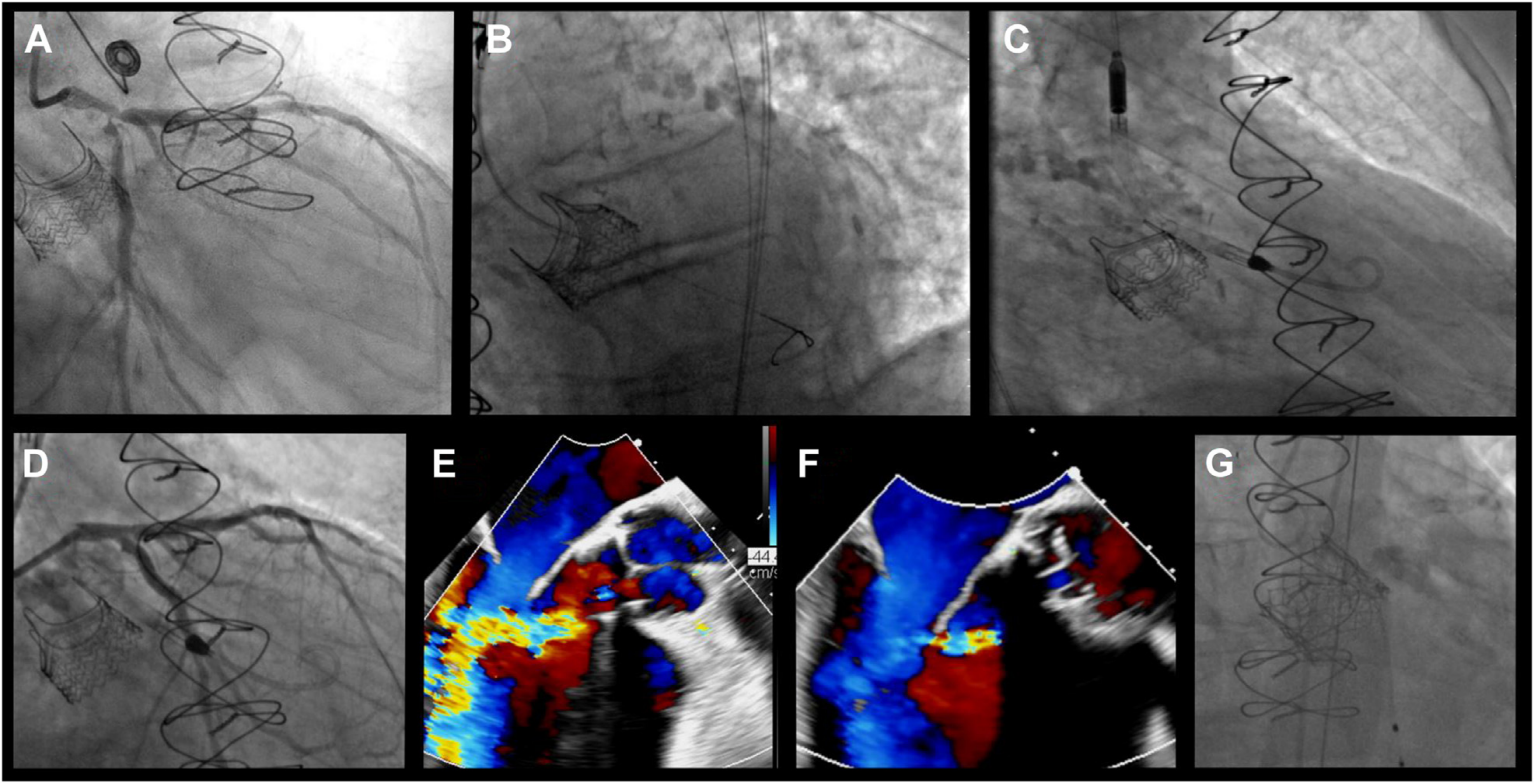
**Angiography and echocardiography imaging of the case.** (**A**) Diagnostic coronary angiogram. (**B**) Left anterior oblique view of the wire into the left ventricle. (**C**) Right anterior oblique view after the Impella was placed into the left ventricle via the paravalvular channel. (**D**) Final angiogram after percutaneous coronary intervention. (**E**) Transesophageal echocardiogram showing severe aortic insufficiency after removal of Impella. (**F**) Transesophageal echocardiogram after valve-in-valve implant. (**G**) Aortic root angiography showing minimal aortic insufficiency.

The patient was a 72-year-old man with a history of aortic stenosis status post-surgical aortic valve replacement (AVR) with a 27-mm Intuity, rapid-deployment, surgical valve (Edwards Lifesciences) and single-vessel coronary artery bypass graft 4 years prior to admission. His other history was notable for at least moderate paravalvular regurgitation since his surgery and functional class III heart failure symptoms. He developed acute onset chest pain and presented to his local hospital. He was diagnosed with a non–ST-segment elevation myocardial infarction and acute congestive heart failure. He underwent left heart catheterization, which revealed closure of the single vein graft with severe left main, left anterior descending (LAD), and left circumflex bifurcation stenosis (Figure 1A). An intra-aortic balloon pump was placed. He was transferred to our institution for surgical evaluation for repeat coronary artery bypass and surgical AVR. An echocardiogram demonstrated an ejection fraction of 25%. The valve prosthesis was not well visualized, with difficulty obtaining quantitative measurements. Moderate-to-severe paravalvular aortic insufficiency (AI) was noted by color Doppler and pressure half-time of 334 milliseconds. Repeat surgical therapy was not offered, as he was determined to be at prohibitive risk. He was then referred for high-risk PCI with Impella support.

At the time of PCI, the intra-aortic balloon pump was exchanged for an Impella CP sheath (Abiomed). A wire was advanced easily into the left ventricle. A femoral right 4 catheter and, subsequently, a 0.018-inch wire were exchanged into the left ventricle without resistance in a left anterior oblique view. The Impella was advanced easily over the wire into an appropriate position (Figure 1B). However, a right anterior oblique view confirmed that the device was actually inserted in a paravalvular position (Figure 1C). With concerns that repositioning would lead to wide-open paravalvular AI, the Impella was left in place and was noted to have satisfactory flow with appropriate ventricular and aortic waveforms. The decision was made to proceed with PCI using a single-access approach. This was completed without complication utilizing a double-kiss crush technique with a 4.0-mm × 33-mm drug-eluting stent (DES) from the left main into the LAD (post-dilated in the left main to 4.5 mm), a 3.5-mm × 26-mm DES to the ostial circumflex, and a 3.5-mm × 18-mm DES to the mid LAD (Figure 1D).

Following successful revascularization, coronary equipment was removed and the Impella left in place. A transesophageal echocardiogram (TEE) revealed only mild AI as the Impella device obstructed flow through the paravalvular channel. The Impella was turned to P0 without significant hemodynamic change. It was easily removed without significant resistance, and no disruption to the existing valve was noted fluoroscopically. TEE now revealed torrential paravalvular AI, with aortic diastolic reversal of flow noted (Figure 1E). A wire was placed through the true lumen of the aortic valve, and balloon valvuloplasty was performed with a 28-mm True Dilatation Balloon (Bard Vascular Inc) in attempts to expand the Intuity valve skirt. The true inner diameter of the Intuity valve was previously noted at 25 mm. Although the valve frame appeared to expand, it recoiled following valvuloplasty, with no significant change in the degree of AI. It was decided to proceed with a valve-in-valve implant (a 29-mm SAPIEN 3 valve [Edwards Lifesciences]), with the goal being to remodel and prevent recoil of the Intuity valve as the Intuity valve cannot be fractured. Given the expansion with a 28-mm True balloon and the paravalvular leak with the 27-mm Intuity valve, we elected to proceed with a 29-mm valve. After deployment of the transcatheter valve, the degree of paravalvular AI did not significantly change. A 28-mm True balloon was then inflated to >20 atmospheres; further stable expansion of the transcatheter and Intuity valve was noted. TEE demonstrated only mild insufficiency, with aortic diastolic flow reversal no longer evident (Figure 1F). Aortic root angiography revealed minimal AI (Figure 1G). All equipment was removed without incident. He was extubated that same day. He required additional diuresis but was ultimately discharged to home on postprocedural day 8. At the 1-month follow-up, he had New York Heart Association class I symptoms. Echocardiography revealed mild paravalvular AI with a mean gradient across the valve of 5 mm Hg and a left ventricular (LV) ejection of 50%.

## Discussion

Nearly 50% of complications with Impella are due to bleeding and vascular site injury. Deployment and retrieval are responsible for 18% of reported complications.^2^ The Intuity valve is a sutureless, stented valve on a balloon-expandable, cloth-covered stainless steel frame. The expandable frame aims to establish a seal across the LV outflow tract. Early and late paravalvular leaks are reported in 1.1% and 1.8% of patients, respectively.^3^ This patient had early paravalvular regurgitation.

Our initial mistake was not confirming wire position in a second view prior to Impella placement. We were fortunate that this improved his hemodynamics by providing LV support while blunting the paravalvular leak. An early valve team discussion led us to complete revascularization to reduce left main ischemia should Impella removal have led to unstable insufficiency. Similar patients have been successfully treated with balloon valvuloplasty alone.^4^ Valve expansion was noted in our patient but proved ineffective in reducing the degree of insufficiency. The transcatheter implant with high-pressure dilation facilitated adequate and stable expansion of the Intuity valve and ameliorated the paravalvular AI.

## Conclusion

Our case demonstrates an unusual complication with an impromptu solution and, fortunately, a positive outcome in a surgically prohibitive–risk, complex patient. This case especially reiterates the importance of contralateral views prior to Impella insertion in patients with a history of AVR, particularly those with AI.
